# A Comment on “Combination of the Fibrosis-4 Index and Carbohydrate Antigen 125 to Predict Morbidity and Mortality in Acute Heart Failure”

**DOI:** 10.31083/RCM50121

**Published:** 2026-04-22

**Authors:** Sefa Tatar, Yunus Emre Yavuz, Ahmet Taha Sahin

**Affiliations:** ^1^Department of Cardiology, Necmettin Erbakan University, Meram Faculty of Medicine, 42090 Konya, Turkey

Dear Editor,

We read with great interest the recently published article evaluating the prognostic value of cardiac biomarkers in patients with acute heart failure [1]. The authors should be congratulated for addressing an important and clinically challenging area, namely the identification of simple, readily available biomarkers that may assist clinicians in risk stratification within a heterogeneous and complex patient population. The focus on combining congestion-related and fibrosis-related indices offers a valuable perspective that extends beyond traditional hemodynamic and neurohormonal parameters.

Acute heart failure continues to be associated with substantial morbidity, frequent rehospitalizations, and high short- and mid-term mortality, despite significant therapeutic advances. Consequently, there is an ongoing need for practical prognostic tools that are not only accurate but also feasible for widespread clinical use. In this context, biomarkers derived from routine laboratory parameters are particularly attractive, as they can be easily implemented without additional cost or infrastructure.

The current study primarily emphasizes morbidity-related outcomes, including clinical deterioration and rehospitalization. These endpoints are of high clinical relevance, as they directly impact patients’ quality of life and represent a major burden on healthcare systems. By focusing on these outcomes, the authors provide important data that may support more individualized follow-up strategies and early identification of patients at risk for recurrent decompensation.

We would like to respectfully contribute to this discussion by highlighting previously published work that may help further contextualize the findings of the present study [2, 3]. In our earlier investigation, published in the *Journal of Geriatric Cardiology*, we evaluated the prognostic significance of the Fibrosis-5 index (FIB-5) in patients hospitalized with acute decompensated heart failure [4]. In that cohort, FIB-5 emerged as an independent predictor of short-term mortality, even after adjustment for established clinical and laboratory variables. Importantly, this association was observed across different heart failure phenotypes, suggesting that fibrosis-related indices may capture systemic pathophysiological processes that transcend conventional classifications based on ejection fraction. The FIB-5 index is a fibrosis-based score calculated using the following formula: FIB-5 = [albumin (g/L) × 0.3 + platelet count (10^9^/L) × 0.05] – [alkaline phosphatase (U/L) × 0.014 + AST-to-ALT ratio × 6 + 14]. In contrast to the Fibrosis-4 index (FIB-4)—which incorporates age, aspartate aminotransferase (AST), alanine aminotransferase (ALT), and platelet count—FIB-5 additionally includes serum albumin and alkaline phosphatase, parameters that may better reflect systemic congestion, hepatic dysfunction, and inflammatory burden in acute heart failure. In a previously published cohort of patients hospitalized with acute decompensated heart failure, FIB-5 was independently associated with short-term all-cause mortality and demonstrated acceptable discriminative performance (C-statistic ≈0.70) [4].

While the recently published article focuses predominantly on morbidity and clinical worsening rather than mortality, these two perspectives appear to be complementary rather than overlapping. Taken together, the findings from both studies support the concept that fibrosis-based biomarkers may provide multidimensional prognostic information in acute heart failure, ranging from short-term survival to subsequent clinical instability. Such a multidimensional approach is particularly relevant in acute settings, where clinicians must simultaneously address immediate prognosis and longer-term disease trajectories.

Fibrosis-related indices such as FIB-4 and FIB-5 are derived from routinely measured laboratory parameters, including liver enzymes, platelet count, albumin levels, and age. These components reflect not only hepatic involvement but also systemic inflammation, congestion, and end-organ interaction, all of which play key roles in the pathophysiology of acute heart failure. Previous studies have demonstrated that abnormal liver function tests and fibrosis-related markers are associated with worse outcomes in heart failure, further supporting their biological plausibility as prognostic indicators.

In parallel, congestion-related biomarkers such as carbohydrate antigen-125 (CA125) have been shown to reflect serosal involvement and systemic volume overload. Elevated CA125 levels have been consistently associated with disease severity, congestion burden, and adverse clinical outcomes in heart failure [5]. The complementary prognostic value of fibrosis-related indices (FIB-4/FIB-5) and CA125 may be explained by shared and interacting pathophysiological mechanisms in acute heart failure. Fibrosis-based indices reflect chronic hepatic injury resulting from prolonged venous congestion, reduced cardiac output, and sustained neurohormonal activation, particularly renin–angiotensin–aldosterone system overactivity. Progressive hepatic fibrosis may further aggravate systemic volume overload by impairing hepatic venous drainage, promoting sodium and water retention, and amplifying systemic inflammation and endothelial dysfunction. In parallel, CA125 reflects mesothelial activation and serosal inflammation triggered by elevated venous pressures and systemic congestion. Together, these biomarkers integrate structural organ involvement and dynamic hemodynamic stress, providing multidimensional prognostic information in acute heart failure.

From a practical perspective, fibrosis-related indices and CA125 may be interpreted using a risk-gradient rather than rigid cut-off approach. Higher FIB-4 and FIB-5 values—particularly those within the upper distribution ranges reported in heart failure cohorts—have been associated with adverse outcomes and may identify patients at increased risk. Fibrosis-based indices, reflecting more chronic organ involvement, may be most informative at admission or early during hospitalization for baseline risk stratification. In contrast, CA125, as a dynamic congestion marker, may be reassessed during hospitalization and in the early post-discharge period to monitor decongestion and residual volume overload. Importantly, serial changes—such as declining CA125 levels with treatment—may reflect effective decongestion and have been linked to improved clinical outcomes, whereas persistently elevated fibrosis-related indices may indicate ongoing systemic vulnerability. These biomarkers may therefore support individualized monitoring strategies, although prospective studies are needed to define validated thresholds and biomarker-guided treatment algorithms. Although universally validated cut-off values specific to acute heart failure are not yet established, available evidence allows a pragmatic risk-gradient interpretation. For FIB-4, values above 3.25—traditionally associated with advanced fibrosis—have been linked to adverse outcomes in heart failure populations, whereas values below 1.45 generally indicate lower risk. For CA125, levels exceeding 35 U/mL have consistently reflected significant congestion and higher morbidity and mortality risk, with higher values indicating greater congestion burden [6]. In contrast, FIB-5 currently lacks standardized thresholds; thus, higher-risk patients are best identified by values in the upper distribution ranges or by persistently elevated levels. Importantly, these biomarkers should be interpreted in conjunction with clinical findings, and prospective studies are required to define heart failure–specific cut-off values. With respect to timing and reassessment, fibrosis-related indices such as FIB-4 and FIB-5 reflect more chronic organ involvement and therefore may be most informative when assessed at hospital admission or early during hospitalization for baseline risk stratification. Routine daily reassessment of these indices is unlikely to provide incremental prognostic value. In contrast, CA125 represents a dynamic marker of systemic congestion and may be reassessed during hospitalization—particularly at discharge—and during early post-discharge follow-up to evaluate the effectiveness of decongestive therapy and detect residual or recurrent congestion. This differential reassessment strategy aligns with the biological behavior of these biomarkers and may enhance their clinical interpretability.

Beyond FIB-4 and FIB-5, other fibrosis-related indices and multimarker strategies have also been explored in patients with acute heart failure. In particular, liver fibrosis–based markers such as the aspartate aminotransferase–to–platelet ratio index (APRI) have been shown to reflect hepatic involvement secondary to chronic congestion and reduced cardiac output, and have been associated with adverse clinical outcomes in heart failure populations. In parallel, several studies have evaluated multimarker approaches combining fibrosis-related indices with established cardiac and congestion biomarkers, including N-terminal pro–B-type natriuretic peptide (NT-proBNP) and CA125, demonstrating incremental prognostic value compared with single-biomarker assessment. These findings support an emerging paradigm in which biomarkers capturing chronic organ remodeling and fibrosis are interpreted alongside markers of hemodynamic stress and systemic congestion. Within this broader landscape, combinations such as FIB-4 + CA125 and FIB-5 should be viewed as components of a wider multimarker strategy rather than isolated prognostic tools, helping to contextualize their clinical relevance in acute heart failure [7].

Several potential confounders should be considered when interpreting fibrosis-related indices and CA125 in acute heart failure. FIB-4 and FIB-5 incorporate liver enzymes, platelet count, and albumin levels, which may be influenced by non-cardiac conditions such as chronic liver disease, hematologic disorders, or medication-related thrombocytopenia. Likewise, CA125 is not cardiac-specific and may be elevated in malignancy, cirrhosis, or inflammatory conditions that can coexist in older heart failure populations. In the original study and in prior FIB-5 analyses, major clinical confounders were addressed through multivariable adjustment, and patients with advanced liver disease or active malignancy were excluded when appropriate. Nevertheless, these biomarkers should be interpreted within the broader clinical context, and future studies may further refine their specificity through sensitivity analyses or subgroup analyses excluding patients with significant non-cardiac sources of biomarker elevation.

From a clinical standpoint, this integrative biomarker approach may help refine risk stratification strategies and identify patients who could benefit from closer monitoring, more aggressive decongestive therapy, or tailored follow-up after discharge. Importantly, the use of inexpensive and widely available biomarkers aligns well with the realities of routine clinical practice, particularly in resource-limited settings.

We believe that acknowledging earlier related studies may help readers better appreciate the evolving role of fibrosis-based biomarkers in heart failure and foster a more comprehensive understanding of how different biomarker pathways can inform prognosis. Such contextualization does not detract from the value of the current study; rather, it strengthens the overall evidence base by situating new findings within a broader and increasingly coherent body of literature.

Looking ahead, prospective studies with larger cohorts and longer follow-up durations may further clarify how fibrosis-related and congestion-related biomarkers can be optimally combined to guide clinical decision-making. Additionally, future research may explore whether biomarker-guided strategies can translate into improved outcomes through targeted therapeutic interventions or structured post-discharge care pathways. We hope that this perspective will be received in the constructive spirit intended and will contribute positively to the ongoing scientific dialogue regarding biomarker-based risk stratification in acute heart failure.

## Abbreviations

FIB-4, Fibrosis-4 index; FIB-5, Fibrosis-5 index; CA125, Carbohydrate Antigen-125.
